# Transcending Ethnic and Religious Barriers in Decision-Making: A Case of a Muslim Women Civil Organisation in Nigeria

**DOI:** 10.3389/fpsyg.2018.02693

**Published:** 2019-01-17

**Authors:** Rofiah Ololade Sarumi, Olumuyiwa Temitope Faluyi, Obianuju E. Okeke-Uzodike

**Affiliations:** ^1^College of Law and Management Studies, School of Law, University of KwaZulu-Natal, Pietermaritzburg, South Africa; ^2^College of Humanities, School of Social Sciences, University of KwaZulu-Natal, Pietermaritzburg, South Africa; ^3^Department of Applied Management, Faculty of Management Sciences, Durban University of Technology, Pietermaritzburg, South Africa

**Keywords:** women, decision-making, politics, civil organisation, Nigeria

## Abstract

Globally, women have more access to positions of authority and participate more in decision-making, regardless of context and rank, than a few years back. This is because of on-going global campaigns supported by various national and international laws and declarations. Increasingly, women have been exercising their rights and obligations to actively participate in politics and become visible in governance. Within Nigerian society, the efforts of women in governance, especially in the pre-colonial era, cannot be overlooked. Over the years, women in Nigeria have moved from the back seat to the roles of bread-winners, decision-makers and leaders of their immediate families. Irrespective of these progressive developments, women's contributions to national development still have minor impact on decisions that affect society as a whole, due to the perceived marginalization which still holds strong in most developing countries like Nigeria. Cultural and social norms, glass ceiling effects, and other exclusionary factors continue to dictate relegation of the presence and voice of women to a lower rank in public life. Against this background, this paper attempts to shed light on the role of civil organizations in enhancing women's participation in politics and decision-making in governance issues in Nigeria. The paper examines strategies employed by a Muslim women civil organization in contributing to the decision-making that affect women generally in the society, and possible challenges facing women active participation in this political era. Using documented researched materials, the findings of the study show that women's intellectual, and political capabilities irrespective of the religious background, are vital components of every society.

## Introduction

Recent statistical reports show that females constitute 49.5% of 198 million people in Nigeria (National Bureau of Statistics, 2016; National Population Commission, 2018; Trending Economics, 2018). The high population of women in Nigeria provides the numerical strength and potential necessary for them to make a positive impact on the country's economic, social as well as political development. Despite the high population of women globally and nationally, the presence of women in decision-making positions in almost all spheres of life is relatively low.

History shows that after the 1960 independence, only about “3.1% of women were elected into political offices and about 5% occupied appointive posts” (Ngara and Ayabam, 2013, p. 47) however, since return to democratic rule in 1999, more women have been elected and appointed into decision-making positions (Luka, 2011). To ensure a more gender-balanced society, the National Gender Policy (NGP) recommends at least 30% representation of women in governance at elective, political and appointive public service positions (Federal Republic of Nigeria, 2008; Oluyemi, 2016). Despite this, Nigeria struggles to meet the recommendation, and has been identified as the country with the lowest number of female representation at both national (6.4%) and sub-regional (11.4%) parliamentary levels in the Economic Community of West African States (Premium Times, 2018; The World Bank, 2018; ThisDay, 2018). A further breakdown of Nigeria's national parliament shows that women occupy 6% of legislative seats in the National Assembly, 7.3% at the Senate and 5.3% in the House of Representatives [Oluyemi, 2016; (PLAC), 2018a]. As a result, Nigeria was ranked 181 out of 183 countries (Inter Parliamentary Union (IPU), 2017) and 133rd in the world, for female political representation (Ogbonna, 2016). Though there is an increase in the number of women in politics in Nigeria [(PLAC), 2018b], the positions they occupy have been mostly appointive as opposed to elective (Ngara and Ayabam, 2013).

Women under-representation in political affairs has been linked to a complex mix of socio-economic factors [(PLAC), 2018b]. Studies have shown the correlation between social factors such as demography (religion, gender, ethnicity, race etcetera) and societal settings to women decision-making (Brunson et al., 2009; Nigatu et al., 2014; Osamor and Grady, 2018). A growing number of researches have shown an unprecedented influence of religion on the low participation of women in politics. Though every religion promotes somewhat different norms, creates different institutions, and builds on different cultural and historical foundations (Klingorová and Havlíček, 2015), studies have shown that religion positively impacts on women decision-making and socio-political position in the society (Njoh and Akiwumi, 2012; Fuseini and Kalule-Sabiti, 2015; Klingorová and Havlíček, 2015). This is very much true for the three predominant religions in Nigeria namely; Christianity, Islam and indigenous African religion (Ojo, 1997; Osamor and Grady, 2018).

This paper specifically focused on the Muslim women organization known as Federation of Muslim Women's Organizations in Nigeria (FOMWAN) and attempts to contribute to the multidisciplinary debate on the influence of demographic factors such as religion and ethnicity on women decision-making and political participation within the society. The researchers' decisions to focus on Nigeria and drawing from a Muslim perspective stem from (a) the high patriarchy system responsible for the meager number of women's active participation in politics (Onyenwere, 2017) and; (b) the small but growing number of studies on the contribution of Muslim women organizations in decision-making and political participation in Nigeria. The paper relies mainly on the analysis of secondary data such as government and non-governmental publications, journal articles, and other published and unpublished materials. Beyond the introductory section, the next section addresses the conceptual issues surrounding decision-making from a feminist perspective. Subsequently, literature on women and decision-making in the multi-ethnic state of Nigeria is analyzed. The study on FOMWAN sheds light on how the organization aligns with the programmes of government to facilitate governance and decision-making and foster national integration. Information on FOMWAN and its strategic activities in transcending ethno-religious barriers in decision-making, as well as limitations to the activities of FOMWAN, lead to the paper's conclusion, and possible recommendations.

## Theoretical Perspective on Feminism and Decision-Making

Historically, the concept of feminism was triggered by the perceived societal structures and subordination limiting women from gaining their full potential. Feminism is influenced by the ideology of historical exploitation, devaluing and oppression of women; criticism of traditional gender ideologies and biases and the drive toward an equal society (Walker and Thompson, 1984; Singh, 2007). The early Marxist and socialist feminists linked gender inequality and women's oppression through capitalist and traditional patriarchy systems evidence in society.

The concern for feminists emerged from the notion of gender equality and promotion of equal rights expressed through theories and actions that devalue women. The developments in the epistemology of feminism have witnessed movements and thinkers such as liberal, radical, Marxist, socialist, post-modern and present day Third-wave feminists. Given the importance of achieving the sustainable development goal of gender equality, this paper is underpinned by the Standpoint theory and influenced by the thinkers of Third-Wave feminism.

The Standpoint theory originated in the early 1970s by Marxist and socialist feminists who were interested in understanding how hierarchical structures such as capitalism and patriarchy, amongst others, influence, shape and limit knowledge practices (Smith, 1974; Harding, 1986, 1993; Collins, 1997). The Standpoint theory was first captured and defined by Harding (2004, p. 1) as “a feminist critical theory about relations between the production of knowledge and practices of power.” The Standpoint theory consists of two schools of thought, the Situated-Knowledge School and the Epistemic Advantage School (Intemann, 2010). According to Wylie (2003), the thesis of situated knowledge influences an individual's experiences, and shapes and limits what the individual knows; such knowledge is achieved from a particular standpoint. In addition, the scholar noted that specific standpoints such as that of a marginalized or oppressed groups, are to a certain degree epistemically advantaged (Wylie, 2003). Standpoint theory is premised on the belief that people from the oppressed or marginalized social group have certain access to knowledge. In a contemporary, demographically stratified society, social positions define to a certain degree what an individual can know. As such, the advocates of this viewpoint posit that knowledge of oppression can be a source of critical insight, and when enhanced efficiently and effectively, it could empower and add to the development of oppositional consciousness (Collins, 1997; Harding, 2004).

Similarly, the Third-wave feminist movement emerged in the mid-1990s as a result of post-colonial and post-modern thinking in feminine discourse. The movement was rooted in the work of early 1980s theorists and coined the term intersectionality by Kimberlé Williams Crenshaw- a scholar of gender and critical race theory (VOX, 2018). The intersectionality addresses the “interlocking nature of identity—that gender, race, ethnicity, sexuality and class never function in isolation but always work as interconnected categories of oppression and privileged” (Henry, 2004, p.32). In addition, the generation of women driving third wave feminism was influenced by their mothers and this generation acknowledged amongst others diversity, change, conflict and contradiction into the movement (Heywood and Drake, 1997; Tong, 2009). The movement focused on the younger generation of women and addressed the concept of feminism in line with mainstreaming gender, recognizing that women are of different colors, nationalities, cultural backgrounds and ethnicities (Guy-Sheftall, 2002; Axelsson, 2015). The concept of Mainstreaming Gender Equality (MGE) supports the notion of assessing the implications of any planned action for both women and men, with the objective of addressing and integrating gender-related issues into all levels of society, politics and programmes (United Nations, 2002; The Commonwealth, 2016). The concept of MGE has been widely embraced and acknowledged as a mechanism geared toward ensuring equality in society and has been well documented in the work of some of the third wave African feminist writers. An example is Chimamanda Ngozi Adichie in her book rooted in inclusion and awareness, “We Should All Be Feminists” (Adichie, 2018). Adichie has not restricted her views to the prints but has extended the movement through audio in series of her TEDx productions (https://www.chimamanda.com). Another feminist writer and researcher driving the third wave feminism is Professor Amina Mama who has written extensively in all aspects of women's involvement in politics, various spheres of work and the economy. She has shown commitment in strengthening activism and activist research in the African context in some of her publications such as “Women's Studies and Studies of Women in Africa During the 1990's” and “Beyond the Masks: Race, Gender and Subjectivity.” In these books, Amina advocate's the need for women liberation in the changing nature of the world drawing most importantly from Africa where traditional belief systems hold strong on the state of women (UCDavis, 2018).

Given the background, the Third-Wave feminism movements advocate the need to accommodate diversity within the ambient of MGE and the changing nature of society (Swirsky and Angelone, 2016). The advocates of this movement embrace culture, ethnicity, religion and race and bring into context the invisibility and impact of such demographic trends in decision-making and their contribution to the development of society. The gender gap in decision-making processes has been widely acknowledged and contested. The struggle for women's voice in social, cultural and economic decision-making processes has continued to gain prominence across all spheres. The Beijing Women's conference of 1995 reaffirmed the gender gap in decision-making and asserted the need to ensure women's equal access and increasing women's capacity to participate in power structures and decision-making (United Nations, 2005). Though progress has been recorded in gender mainstreaming lately, the pace of change is slow, uneven, and very low in all aspects of life. Women's participation in decision-making is relatively low compared to the increase in the population of women in various countries around the world.

## The Multi-Ethnic Nature of the Nigerian State

The concept of ethnicity relates to common values, beliefs and practices (Smith et al., 2009). Ethnicity in the Nigerian context relates to the common identity and affinity which a group of people have. Such groups have ascribed membership, usually based on claims of myths of common history, ancestry, language, race, religion, culture and territory (Osaghae, 1995; Ukiwo, 2005). These shared features and traits become the basis for differentiating group from others and stipulate the standards, values and criteria upon which people act and make decisions (Osaghae, 1995). Nigerians hold ethnic and religious affiliations in high esteem; as such, members of one ethnic group view other ethnic groups as different from them. This is why Osaghae (1995, p. 11) defined ethnicity as “the employment or mobilization of ethnic identity and difference to gain advantage in situations of competition, conflict or cooperation”. Similarly, (Ayatse and Akuva, 2013, p. 180) assert that ethnicity is the deliberate and conscious tracing of one's identity to a particular ethnic group, and allowing such feeling to determine the way one relates with people and things around it. Ethnicity thus becomes a phenomenon accorded attention in a multi-ethnic society, as a result of interactions between ethnic groups within the same political system (Ikpe, 2009). This is why ethnicity is a political problem in most multi-ethnic states (Ikpe, 2009). Similarly, religion as a component of ethnicity is defined in literature as “the belief in spirits” or “the belief in the supernatural” (Horton, 1960, p. 201). The concept deals with one's relationship with a super or higher being. Nigeria has three major religions, Islam, Christianity and Traditional religions. The adherents of the traditional religion are the fewest, while various demographical estimates have set the number of Christians and Muslims at almost the same number (Pew forum, 2010).

Nigeria is a country characterized with diversity in ethnicity, language and culture and the country has more than 250 ethnic groups (Attah, 2011; Central Intelligence Agency, 2018). A view into the religious and ethnic strata of Nigeria is germane. The Core Northern part of Nigeria is predominantly made up of the Hausa-Fulani ethnic group who are mainly Muslims. The Middle belt is a conglomeration of minority groups as well as Muslims and Christians, while the South Western part, the Yorubas is dominated by Christians and Muslims. The South Eastern part is mainly of the Igbo ethnic extraction and mainly Christian, while the South-South is a cluster of minority ethnic groups and mainly Christians. Relationships between and among these groups have always been frosty and this has culminated in series of ethno-religious crises in Nigeria. This is because the religious and ethnic identities which Nigerians accord to themselves over-ride national identity (Okpanachi, 2009). Thus, divisions along ethnic and religious lines in Nigeria have created a gulf in inter-group relations, resulting in high record of inter-ethnic and religious conflicts (Okpanachi, 2009; Eniola, 2010; Attah, 2011; Sulaiman, 2015).

In order to mitigate the effects of these ethnic and religious challenges, decisions are made based on ethnic and religious considerations. This explains why Nigeria has affirmative action programmes like Federal Character Principle, National Youth Service Corps, and the appointment of ministers from each state of the federation. However, these have not mended the ethno-religious cracks in the Nigerian state. Political class has always disunited Nigerians through mobilizing support by playing on the ethnic and religious differences of the populace. The effects of these are obvious in the various inter-ethnic, intra-ethnic and religious crises that have bedeviled Nigeria for decades. These include crises such as those of Kafanchan in 1987; the Zango-Kataf-Hausa conflict of 1992; the Ife-Modakeke feud; the Yoruba-Hausa conflict in 2001 in Lagos and Shagamu; the incessant Plateau crisis between Hausa/Fulani and the indigenes; the Maitatsine religious crisis in the 1980s, and the 1990 Muslim-Christian violence in Kano, among others (Alanamu, 2005; Eniola, 2010; Attah, 2011; Meredith, 2011). In addition, literature has documented recent conflicts involving the Fulani Herdsmen and farmers/host communities in various states in Nigeria (Okereke, 2012; Kasarachi, 2016; Ishola, 2018). These violent crises are always perpetrated based on communal, political and economic reasons, which affect the decision-making within the public sphere.

## Women and Decision-Making: A Perspective From Nigeria

The basic functions of the Nigerian government are encapsulated in its 1999 Constitution. Section Women and decision-making: A perspective from Nigeria of the Constitution empowers the government to make laws for the peace, order and good government of the country, while section 13 also states the primary functions of the government as the provision of security and welfare (Federal Republic of Nigeria, 1999). The activities of government revolve around all these, which are implemented through the tripod of policies, programmes and decisions. Salient decisions are made by government in order to allow it deliver its mandate to the people. Such decisions involve the input of other stakeholders such as the private sector, civil society organizations and international partners.

Eneanya (2009, p. 104) defined decision-making as “choosing between alternatives.” Succinctly, the objective of the decision-maker is not to solve all societal problems but some, and there are costs and benefits to be taken into consideration while making decisions. These reveal that a choice or choices is to be made among alternatives. The types of decisions to be taken are consequent upon the objectives of the decision-maker, the characteristics of the person making the decision, the resources available and the environment where such decisions are made (Eneanya, 2009).

While there is no gainsaying on the important responsibilities which women performed in the pre-colonial era, one can also not overlook the contributions of Nigerian women in the post-colonial era. Women like Funmilayo Ransome Kuti, Magaret Ekpo, and Hajia Gambo Sawaba laid the foundations for the emancipation of women at different levels in their societies (Asaju and Adagba, 2013). These women show that over the years, the role of women has evolved from the traditional roles and back seat position which they took in the old, patriarchal society, to the roles of bread-winners, decision-makers and leaders of their immediate families (Onwubiko, 2012). Nowadays, women are positioned to perform more relevant roles in their societies as many are now more educated, economically liberated and knowledgeable (Carli and Eagly, 2001). Women seem to be more equipped to perform important functions and responsibilities within the home, family, business, community and society in Nigeria (Agbalajobi, 2010). More women have taken on roles of activists for various causes and their involvement in politics cannot be overlooked as more women have contested to be elected as members of the Senate and House of Representatives more than ever in the history of Nigeria.

However, it could still be argued that although advancement in education has improved the societal status of women and their number in politics, the visibility of women in decision-making is still obscured due to socio-economic inequalities, illiteracy, ignorance and gender-based violence (Carli and Eagly, 2001; McGarvey, 2009; Faiz et al., 2017). The contributions of women to governance still appear to be insignificant when compared to the contributions made by men.

## An Overview of FOMWAN

FOMWAN is a faith-based organization and an umbrella body for all Muslim women's groups in Nigeria. It was founded at a conference of Muslim women in 1985. The aim of establishing the organization at the time was to restore the Islamic identity of the Muslim woman and to re-establish the leadership status which women enjoyed in the pre-colonial era, especially during the era of Sokoto caliphate (Uthman, 2009). At the time of its formation, FOMWAN was charged with the duty of coordinating the activities of Muslim women's organizations in Nigeria. The main objectives of the FOMWAN include the promotion of unity, cooperation and common action among the Muslim women's groups in Nigeria; the encouragement and coordination of the development of Islamic education and awareness among women, and enabling Muslim women to express their views on national issues.

FOMWAN has the mandate to care for the social and religious needs of women and children particularly, and to promote human rights and development generally. It mobilizes “women to play active roles in all aspects of life, promoting their solidarity and uniting Muslim women organizations in the country to speak with one voice on national issues” (Uthman, 2009, p. 250). In this regard, it engages in programmes which influence decisions of the government on general governance matters, issues which relate to Islam, women, children and the society in general.

Over the years, FOMWAN has met many of the social and civic needs of women through effective social work. It has achieved the task of propagating Islamic education and awareness among Muslim women throughout Nigeria and it continues to provide an opportunity for expressing the Islamic perspectives on children and gender issues (FOMWAN, 2018). FOMWAN promotes the understanding of the position of women and children and other aspects of society at all levels of government. As a civil-society organization (CSO), it engages in social and charity work which includes the establishment of orphanages, model schools and the provision of several other social welfare packages for women, children and the poor and needy in the society. In addition to these, FOMWAN promotes the political, economic and social growth of the individual member groups by engaging in income-generating and capacity-building training.

The membership FOMWAN is open to all Muslim women's organizations in Nigeria and the membership spans across all the 36 states of Nigeria. Thousands of women's organizations in Nigeria have registered their organizations with FOMWAN. Since the establishment of FOMWAN, similar women's organizations have sprung up in other countries like Ghana, the Gambia, Liberia, Mauritius, Niger, and Sierra Leone (FOMWAN, 2018). The members of the individual women's associations meet through their regular group meetings known as “*Asalatu*” (these are meeting sessions where Allah is remembered, and the teachings of Islam, as well as societal issues, are discussed). Thus the meetings are used to educate women on other aspects of Islam as well as contemporary issues which affect women and the society. The state umbrella body meets fortnightly and the national headquarters of FOMWAN organizes quarterly meetings and annual national conferences at the national level. These annual conferences provide opportunities for women to discuss national issues which affect women as individuals within their societies and it allows the state arms of the organizations to relate with each other, network and to share best practices.

FOMWAN has long recognized the fact that women have an important role to play in societal and national development. The organization believes that the scope of women's contributions to national development “goes beyond the narrow customary conception of their role in family keeping and procreation and permeate all facets of the nation's economy” Coleman in Onwubiko (2012, p.68). Over the years, FOMWAN has established itself as a reputable non-governmental organization (NGO) and has earned high credibility within the Nigerian civil society. It performs many of its social work through charity, donations and other sources of funding. It has over the years participated in decision-making activities, debates, conferences and has partnered with the national and state governments to work on matters which are in the interests of the public.

## Bridging the Barrier: The Case of FOMWAN and Decision-Making in Nigeria

The advancement of women into decision-making roles has taken center stage and forms one of the key objectives of many countries' policies. Recently, the empowerment and visibility of women in society has been acknowledged more than ever in the history of mankind. Increasingly, women are gaining more access to positions of authority and leadership at varying degrees and within the country's perspective. The positive mileage in women's decision-making roles can be attributed to factors such as institutional missions, structures and capabilities (Markham, 2013; O'Neil and Domingo, 2015). O'Neil and Domingo (2015), assert that while institutions address the rules and norms that shape people's behavior and interactions, structures are the deeper social, economic and political endowments, groupings and patterns that shape a society. In addition, Markham (2013: p. 10), defines “institutional structures as the country's constitution, the electoral system and the legal special measures (such as a gender quota, if they exist).” Therefore, women take advantage of the available institutions and structures to empower and improve on the capacity they are given, for example, from education (O'Neil and Domingo, 2015).

Narrowing down to FOMWAN, the organization is currently affiliated to over 500 secular national and international organizations and bodies. The organization has been able to transcend the ethnic and religious divisions which have plagued Nigeria over the years. This is due partly to the fact that membership of FOMWAN is made up of women from different ethnic, educational, social, cultural and economic backgrounds in Nigeria. It is also significant that the membership of FOMWAN is of both the highly educated and successful as well as the uneducated, those on the lower rung of the socio-economic ladder. This results in a wider inclusion of women. The congregation of women from all the geopolitical zones in Nigeria, with different capabilities, in different fields, adds to the strength of the organization because of the wealth of competencies it brings together.

FOMWAN as a CSO is not directly involved in politics; nevertheless, Lynn has affirmed that politics has strategic importance for women because the ultimate success of women's movements will rest heavily on effective use of the political process (Lynn, 1978). FOMWAN's interactions with other secular NGOs within the civil society have direct and indirect influences on governance and decision-making. It has positioned itself strategically through the reputation which it has carved for itself over the years as a major stakeholder in women and children's affairs specifically. It has also participated in affairs other than those of women and children. Thus, the organization is usually invited to governmental debates and discussions, and evidence shows that the opinions of FOMWAN have usually been taken into account when important governmental decisions are made. Examples of such cases where the viewpoints of FOMWAN have influenced government decisions, include when it was called to clarify the Islamic viewpoint on Islamic marriage when discussions on the eradication of child marriage were being held by the government (Uthman, 2009). Other instances include seeking its opinion on the Islamic views on family planning, female genital mutilation (FGM) and the elimination of other religious and cultural practices which are harmful to women and children (Uthman, 2009).

The unity and cooperation within FOMWAN, which is founded on religion, has helped the organization to rise above all other divides which exist in Nigeria. The inter-state and inter-ethnic interactions and cooperation are invaluable in Nigeria, considering the very fragile state of the country and the conflicts fuelled by the differences among the various ethnic and tribal groups in the country. This is, therefore, an invaluable contribution to the much-needed unity and cohesion among the diverse ethnic groups. FOMWAN has been able to use this unity and cooperation to positively impact the society. For instance, FOMWAN's involvement and advocacy on education have been very productive at the state and national levels. In previous years, FOMWAN dedicated its education programme to address the problems relating to education in Nigeria. In fact, the theme of its 27the National Conference in 2011 was “Education: A Tool for National Development,” aimed at drawing government attention to the issues of great national importance (Matazu, 2011). The organization also hosts an Education Summit annually to which they invite reputable academics, educators and government officials. Some of the recommendations made at these summits have been complementary to the government's efforts aimed at improving the education system in Nigeria. Some of the efforts have also been directed at curbing the menace caused by the “A*lmanjeri*” system of Education in Northern Nigeria. The A*lmanjeri* system of education is where students (predominantly males) are sent off to seek knowledge while living with a teacher who teaches them about the Islamic religion. The pupils usually have no financial support so they resort to begging for alms and they perform menial jobs for survival. It is believed that these students become a menace in the societies in which they live, as they have no formal education, very little means of survival and they are idle most of the time. A report also shows that there are more than 10 million A*lmanjeris* in northern Nigeria (Taiwo, 2013).

FOMWAN does not have a mandate to venture into politics; however, the organization has been a platform for Muslim women to make contributions to governance and to participate in decision-making by providing an opportunity for promoting the role which women are expected to play in decision-making. The organization has set a high bar for recognition, relevance and trust, and has shown its open-ness to supporting government efforts which are aimed at empowering women. In this regard, FOMWAN accepts nominations and appointments to specific governmental boards. The nominations and appointments are not limited to boards which have religious mandates alone; on these boards, they act as stakeholders and their services are rendered in the interests of the public. For example, members of FOMWAN have often been nominated as members of the Muslim wing of the Pilgrims Welfare Board. It also partners with the government on initiatives which are aimed at advancing the socio-economic status of women in society.

The organization is also known to have participated actively in monitoring election and voting processes. In fact, FOMWAN was one of the women's Muslim organizations which moved from “passive to active roles in reformist coalitions” by joining the election monitoring coalitions in 2003 (Kew, 2016, p. 337). FOMWAN also served as an observer group during the 2011 elections and was one of the four CSOs which made up the Project 2011 Swift Count (Adesina, 2011). The observer group brought together the election observation skills and legal expertise of Christians and Muslims organizations and secular NGOs during the election. In this regard, the organization has established itself as a significant observer group in Nigeria.

While direct involvement in politics is yet to be explored by FOMWAN, the increasing need for women's participation in politics has become evident. It is clear “from Nigeria's experience that the political process is male-dominated and men influence the process more than women” (Quadri, 2015, p. 4). FOMWAN uses its structure and numerical strength as the umbrella body of all Muslim women's organizations to influence the representation of women in the political process. In addition, FOMWAN's strategic partnerships with other women's organizations has resulted in FOMWAN's resolution to shift from the passive role which most women's organizations initially played, to a more active role within the society. Such is evidenced by its democratically structured coalitions with the National Council of Women's Societies (NCWS) (Kew, 2016) and a partner organization of Gender and Development Action (GADA). This is also indicative of the benefits which FOMWAN can add to such partnerships when they collectively advocate for issues of common concern (Kew, 2016). An example of such matters of common concerns which women's organizations have dealt with collectively, includes the “Bring back our girls” campaign which was held to call for the release of the girls who were kidnapped from their school by Boko Haram in Northern Nigeria in April 2014 (Bringbackourgirls., 2014). The fact that FOMWAN has a good relationship with other secular as well as Christian women's organizations, is also advantageous, as this would ensure the number and representation which are necessary to ensure visible women's representation in governance and decision-making.

In an attempt to positively influence decision-making, FOMWAN has developed some activities which target general society, irrespective of the ethnic or religious preference of the people. Some of the activities which cut across the board include a radio program of FOMWAN, Oyo state branch, called “*Iwa lesin”* which is broadcast on Radio Nigeria 93.5fm every Friday. The scope of this radio program goes beyond religion; it is aimed at encouraging the people to “be the change they want to see” (a Gandhian perspective) in the society. The radio program has audiences across a wide range of age, gender and religions, as well as different socio-economic groups.

FOMWAN also takes its human rights promotion and protection mandates seriously, and advocates for the right to education, children's rights, gender-related rights, sexual and reproductive health rights, as well as several socio-economic rights. The organization is known for taking a front-line stance while advocating for the recognition of the rights to education, inheritance and age of consent to marriage, spousal maintenance as well as the sexual and reproductive health rights of women. FOMWAN pursues its human right mandate in the interests of the public and the impact/benefits of such efforts are enjoyed by Muslims and non-Muslims in the society. FOMWAN also has representatives who sit on various human rights committees within the governments of their various states. This is evidenced in the involvement of FOMWAN, Oyo State in the Child Protection Network, (CPN) and its active contributions to the protection of the rights of all children. FOMWAN as an NGO also engages in awareness-raising and advocacy work through its various internal committees. The organization has various committees which are responsible for enacting several of the themes.

The efforts of FOMWAN have had much influence on the decisions of the government at various levels in Nigeria over the years. The organization has employed various strategies to maintain an active position in decision-making. Such strategies include the use of structures such as an organization for Muslim women; formation of strategic alliances with other civil organizations; partnership with government; good working relationship irrespective of creed, establishment of branches across the nations and maintaining a strong national body. Furthermore, FOMWAN maintains its roles in human rights advocacy, ensuring the mainstreaming of gender, creating educational awareness, and accepting nominations into government boards.

## Limitations, Conclusion and Recommendations

Recent developments have shown that women around the world make decisions that affect their lives, community, and society in general. It can be concluded that FOMWAN has used factors such as institutions, structures and their capabilities, successfully, to address economic and social changes within the Nigeria context. Religion as a phenomenon which relegates women below men, has over the years hindered women from performing beyond the permitted scope. Thus, FOMWAN as a female organization in Nigeria “represents hope for the regeneration of the decayed status of Muslim women in Nigeria” (Uthman, 2009, p. 262). The organization has been able to transcend many of the limits carved by the patriarchal society and in so doing, contributed to placing women in the positions where their words matter. It has contributed to helping women from all spheres of life to come together, unite and fight against subjugation in various facets of the society. As Muslim women make leadership in-roads into all fields in Nigeria, they are learning to devise approaches in maintaining Islamic decorum and eliminating practices harmful to women (Uthman, 2009). FOMWAN has been active in decision-making and the efforts of the organization have multiplied effects in Nigeria irrespective of the plurality of the Nigerian state. Regardless of religious and ethnic factors, FOMWAN has cooperated with the government and contributed to decisions that positively affect Nigeria citizens. The organization has maintained a front-line position in ensuring that women participate actively and contribute positively to society. In addition, from the Standpoint theory perspective, FOMWAN has shown that knowledge is indeed embedded in the oppressed and marginalized groups such as women and such knowledge when effectively harnessed could foster societal development.

Despite FOMWAN's contributions and achievements in many facets of society, it is still limited by the patriarchal nature of traditional Nigerian society, as women are still being relegated to the lower rung of the society in some cases (Uthman, 2009). The influence of religion on the interpretation of the roles of women in society, also limits the scope of the activities in which the members can participate. While FOMWAN is bound by the dictates of the Islamic religion which recognizes the authority of men over women, the organization recognizes that the privileges of the males should not over-ride the God-given rights of females (McGarvey, 2009). Thus, FOMWAN's approach to the interpretation of the roles and rights of Muslim women and women in general, is based on the fact that the rights of women “should not be based on the whims and caprices of male chauvinists who seem to derive pleasure in oppressing women after jettisoning Islam and using culture as a façade” (McGarvey, 2009, p. 181). In addition, many of the activities of FOMWAN are dependent on the availability of funds because the organization relies heavily on donations and the available human resources.

In order to contribute to the visibility of women in the decision-making process in Nigeria, this paper recommends that FOMWAN should:

i. Continue to maintain and encourage the coalition of women and other civil organizations, to ensure that women have one voice on political and governance matters in Nigeria.
ii. Support and advocate for the mainstreaming of women and women's issues into politics, so that the recommendation of the NGP of 35% affirmative action for women in the governance process is achieved.
iii. Continue to use its links across all levels of society to coordinate support and advocate for fellow women aspirants.
iv. Facilitate and contribute to the elimination of factors such as poverty and other socio-economic inequalities, illiteracy, ignorance and gender-based violence, which limit the involvement of women in governance and politics.

## Author Contributions

RS contributed immensely to the development of the first draft of the manuscript. OF's contribution is well acknowledged in the entire manuscript but mostly reflected in the sections on The Multi-ethnic Nature of the Nigerian State and Women and Decision-making: A Perspective from Nigeria. OO-U developed the abstract and the section on Theoretical Perspective on Feminism and Decision-making, she also did the general review and revised the final version of the manuscript.

### Conflict of Interest Statement

The authors declare that the research was conducted in the absence of any commercial or financial relationships that could be construed as a potential conflict of interest.
